# A Scheme with Acoustic Emission Hit Removal for the Remaining Useful Life Prediction of Concrete Structures

**DOI:** 10.3390/s21227761

**Published:** 2021-11-22

**Authors:** Tuan-Khai Nguyen, Zahoor Ahmad, Jong-Myon Kim

**Affiliations:** 1 Department of Electrical, Electronics and Computer Engineering, University of Ulsan, Ulsan 44610, Korea; khaint@mail.ulsan.ac.kr (T.-K.N.); zahooruou@mail.ulsan.ac.kr (Z.A.); 2 PD Technology Co., Ulsan 44610, Korea

**Keywords:** acoustic emission, deep neural network, hit removal, long short-term memory, one-class support vector machine, remaining useful life, stacked autoencoder

## Abstract

In this study, a scheme of remaining useful lifetime (RUL) prognosis from raw acoustic emission (AE) data is presented to predict the concrete structure’s failure before its occurrence, thus possibly prolong its service life and minimizing the risk of accidental damage. The deterioration process is portrayed by the health indicator (HI), which is automatically constructed from raw AE data with a deep neural network pretrained and fine-tuned by a stacked autoencoder deep neural network (SAE-DNN). For the deep neural network structure to perform a more accurate construction of health indicator lines, a hit removal process with a one-class support vector machine (OC-SVM), which has not been investigated in previous studies, is proposed to extract only the hits which matter the most to the portrait of deterioration. The new set of hits is then harnessed as the training labels for the deep neural network. After the completion of the health indicator line construction, health indicators are forwarded to a long short-term memory recurrent neural network (LSTM-RNN) for the training and validation of the remaining useful life prediction, as this structure is capable of capturing the long-term dependencies, even with a limited set of data. Our prediction result shows a significant improvement in comparison with a similar scheme but without the hit removal process and other methods, such as the gated recurrent unit recurrent neural network (GRU-RNN) and the simple recurrent neural network.

## 1. Introduction

Concrete structures are now a very familiar sight due to their features and the ready availability of their materials. Concrete houses, concrete towers, concrete bridges, and numerous other structures have become a fundamental part of the modern day. Concrete has been proven to be a durable material which can withstand even extreme conditions [1], such as fire, weather hazards, and chemical attack. An example of this can be seen in the Pantheon, which has remained in marvelous condition after almost 1900 years. Structural concrete requires reinforcing bars (rebars) to support the bending and torsional loads. Although the rebar offers superior tensile strength and is an essential part, it lowers the lifetime of concrete significantly due to rusting and crack problems. Therefore, it is essential to monitor in-service structures.

Among concrete structures, the beam is one of the most important subjects in structural health monitoring (SHM) topics, as its function is to support loading from above and to withstand compression and tension. For investigating concrete beams, visual monitoring is often used as the most basic technique; however, it cannot be efficiently performed if the location is not accessible/visible. Many studies have been proposed with different non-destructive approaches: the image processing technique [2,3,4], ultrasonic [5,6,7], vibration [8,9,10], acoustic emission [11,12,13,14], etc. Among them, the prominence of acoustic emission (AE) techniques has increased in recent years as a solution for concrete monitoring and for general prognosis and health management (PHM) frameworks. By definition, acoustic emission is the elastic energy internally released by materials when a discontinuity appears. In comparison to other non-destructive techniques, acoustic emission testing is non-directional and little to no downtime is required during in-service testing. Moreover, this method is also superior in terms of being capable of dynamic progression tracking of the discontinuity and evaluating the significance of the deterioration in a single test. The recorded events have a now-or-never quality [15], which means that if an event of discontinuity is not detected at the moment of its occurrence, it would not be available at any other time. However, with consistent improvements in data recording and processing, this problem can be approached with ease. The notable downside of acoustic emission testing is that it cannot detect an existing discontinuity, but given its foremost application of progression monitoring, this is not of interest, as the specimen’s initial condition should be fully investigated before performing an AE test.

Service life can be extended significantly if failure is detected and a RUL prognosis is made in an early stage and the structure is correspondingly maintained. For this purpose, multiple authors have addressed the cracking phenomenon [16,17,18,19] in ongoing studies. Along with these studies, a number of fault detection and diagnosis (FFD) techniques [20,21] have been proposed in recent decades with the aim of prolonging service life. While diagnosis deals with current and past faults, what we might look for in prognosis is how the accumulated faults might affect the specimen in the future. The remaining useful life (RUL), also known as the prognostic distance or lead time, is a crucial metric used in prognosis. By definition, it is the time from when the specimen is under inspection to the moment its useful life ends [22,23]. In general, RUL prediction methods can be roughly sorted into two different approaches: model-based and data-based.

While the model-based approach relies on establishing a mathematical model to imitate a real-life process, which can be exponentially hard with more sophisticated models, the data-based approach is centered around capturing the fault patterns with available data. Without having to investigate both the nature of the system and the fault, the second approach is significantly less complicated, and therefore more favorable in the latest studies [24]. The data-based approach is our primary focus in this study.

With the bloom of machine learning techniques in recent years, the data-based approach has gained numerous benefits [25] compared with the earlier days when statistical methods and principal component analysis were still state-of-the-art approaches. Recent studies concerning AE-based RUL prognosis have been presented with promising results: the authors in [26] suggested the use of adaptive non-homogenous hidden semi Markov model to predict life of composite structures, while those in [27] and others in [28] respectively proposed the use of support vector machine regression and Gaussian process regression for slow speed bearings, etc. With the success of recent deep learning techniques, which have been widely adapted to many areas [25], automatic feature extraction and direct mapping from raw input to output classes have demonstrated promising competency. Our study proposes the use of direct construction of health indicator (HI) lines from raw inputs using a deep neural network (DNN), which is fine-tuned after a stacked autoencoder (SAE) pre-train. Because the number of AE hits during a period of time, which are detected with a constant false-alarm rate (CFAR) [11], a neural network can portray the severity of the deterioration, as it harnesses the input data labels for the training. These HI lines are then used in a long short-term memory recurrent neural network (LSTM-RNN) for HI prediction. The LSTM-RNN is utilized rather than a conventional RNN due to its ability to store and process long-term dependencies, as the data in this study can be considered a long-term time series [25]. From the predicted HI lines, the RUL can be computed accordingly.

In order to provide a closer RUL prediction, it is essential to improve the hit detection process, which is representative of the specimen’s deterioration severity. When a specimen is fractured, its structure is altered. During a time of high AE intensity and with small test specimens, an AE event might cause more than one hit in a sensor (due to reflection, hit spit, refraction, etc.) [29,30] which, with the accumulation over time, can affect how the total number of hits affect the portrait of deterioration severity. There are methods proposed to address such problems, for instance, the two time-driven parameters hit definition time (HDT) and hit lockout time (HLT). However, given that these methods are hard-threshold based, they are susceptible to stochastic phenomena, which can be used to describe the concrete fracture process [29]. Consequently, these pre-set parameters will induce errors, even with careful instrument calibration. In these cases, the implementation of a hit removal process is very important. In our study, we focus on identifying the anomalous hits that differ the most from others through one-class support vector machine (OC-SVM) [31,32].

The following contributions are proposed in this study:

1. A novel hit removal process is proposed using OC-SVM for a better portrait of deterioration process, which has not been investigated in other studies to the best of the authors’ knowledge.
2. The construction of HI lines from raw data through a SAE-DNN with AE hits detected with the CFAR as the training label.
3. RUL prediction based on the LSTM-RNN which outputs HI prediction from the lines constructed in an offline process.

The arrangement of the following sections is as follows: Section 2 provides the experimental setup along with a description of the dataset; the methodology is discussed in detail in Section 3; Section 4 shows the results of our method, with discussion, which is followed by concluding remarks, along with a discussion of future research directions, provided in Section 5.

## 2. Experimental Setup and Dataset Description

### 2.1. Experimental Setup

**Figure 1:**
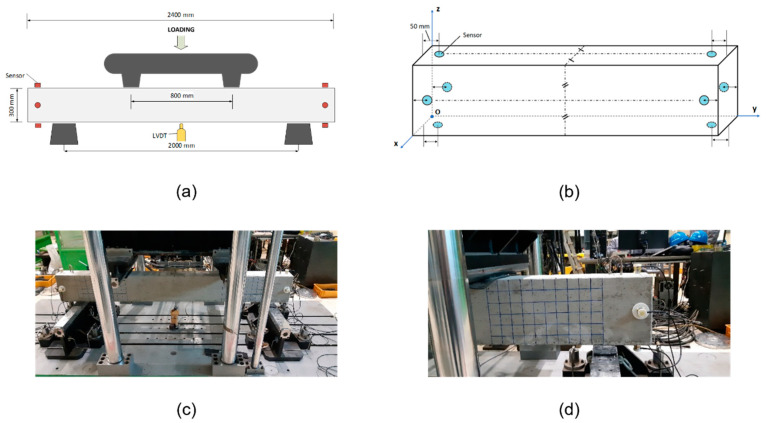
Illustration of the RC beam under a four-point bending test: (**a**) Loading and LDVT placement, (**b**) sensor placement, (**c**) pictorial of the test setup, (**d**) pictorial of the gridded specimen.

In our study, reinforced concrete (RC) beams were utilized for four-point bending tests. We similarly fabricated multiple specimens and gridded them with 50 × 50 mm^2^ squares to improve visual inspection of the fractures. Eight R3I-AST sensors were equally divided into two batches and mounted towards the ends of the beam surfaces for data acquisition at a sampling frequency of 5 MHz. During each test, the specimen was loaded with two concentrated loadings which were positioned 800 mm away from each other. The vertical displacement was measured by a linear variable differential transformer (LVDT) placed at the center of the bottom surface. The experimental setup is illustrated in Figure 1.

The detailed information of this experiment is listed, in Table 1:

Each test was performed until the point when the cracking severity was high enough; however, the collapse of specimens was not allowed to happen considering the lab safety. The entire loading process was monitored by our team and specialists in the construction field.

**Figure 2:**
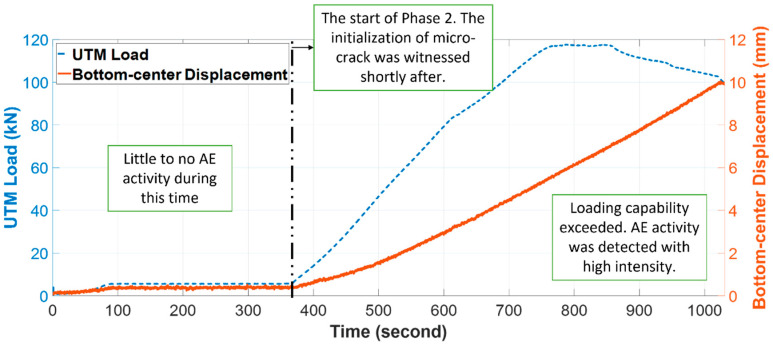
Loading versus the bottom displacement during the two testing phases.

The testing is divided into two main phases: in the first phase, the loading is low and within the specimen’s capability, little to no AE activity is witnessed during this time; in the second phase, the loading increases steadily, and AE activity is present with a very high intensity towards the end. It can be noticed that near the end, even when the universal testing machine (UTM) loading decreases, the displacement of the bottom-center is still aggravated. This behavior is due to the felicity effect that extends to the situations not covered by Kaiser effect, in which AE activity still occurs, even when the current loading is below the previous maximum [15]. It also appeals to the Dunegan corollary [15], which states that if acoustic emissions are observed prior to a previous maximum load, then, at least, a new discontinuity has occurred. An illustration of the two phases is shown in Figure 2.

### 2.2. Dataset Description

The previous section explained, in detail, how the monitoring tests were conducted. In each process, a RC beam with eight equipped sensors underwent the four-point bending test. Data from the three tests are utilized for the validation of our method, which offers a total of 24 run-to-failure streams. These streams are then distributed equally into two sets, with one containing signals from sensors 1 to 4 and the other containing the rest. The signal length of three tests on concrete beams A, B, and C are 600, 650, and 620 s, respectively.

## 3. Methodology

Prior to the description of the methodology, we need to clarify some definitions. An AE event is the release of elastic energy when the subject undergoes deformation. A hit can be considered as the way a transducer (in this circumstance, an AE sensor) perceives the event from its point of view. In this paper, we will discuss events and hits only from AE view, hence from here, an AE event is referred to as “an event” only, just as “a hit” represents the term of an AE hit.

**Figure 3:**
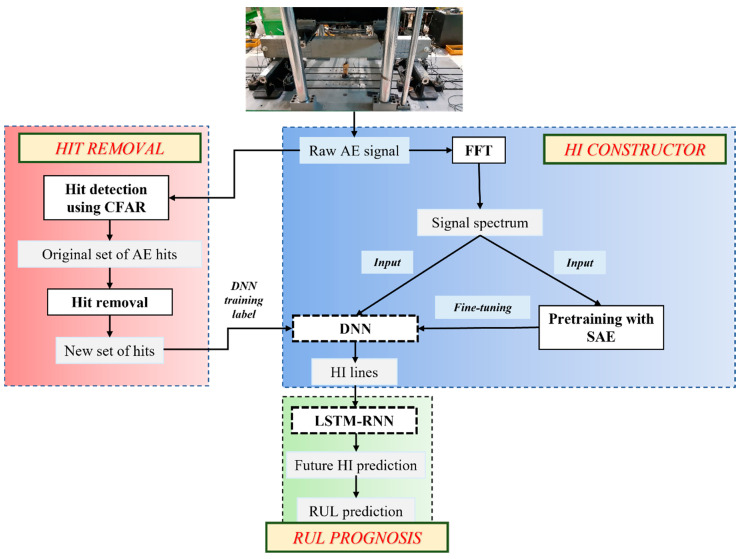
Flowchart of the proposed scheme.

The general framework of our proposed approach is shown in Figure 3.

### 3.1. Hit Detection Using CFAR

The process starts with hit extraction from data, which is done using the average constant false alarm rate (CFAR). The CFAR is an algorithm first introduced for target detection in radar systems. Due to its ability to solve real-life problems in which the noise is usually colored and its variance is often unknown, CFAR can be imported to various applications, one of which is hit detection.

**Figure 4:**
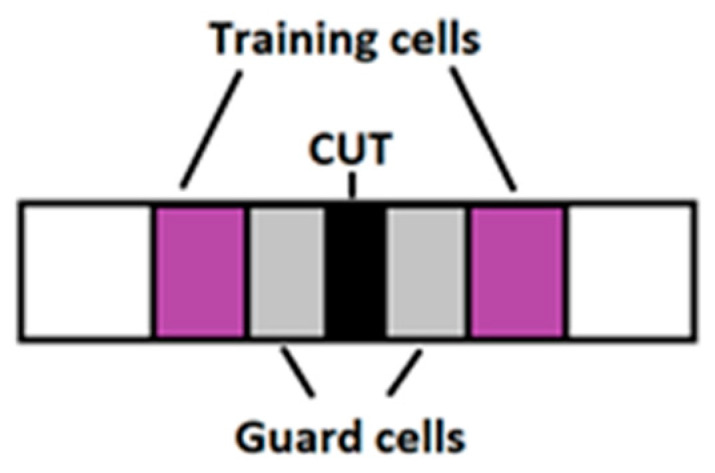
CFAR diagram.

Since the recorded signal is one-dimensional, the model for the CFAR can be seen in Figure 4.

The training cells are protected by the leading and lagging guard cells, which prevent signal components from leaking into them. When a given cell is required to be checked, the noise power is then estimated from its neighboring cells. The threshold for this cell under test (CUT) can be then calculated as follows:

(1) $$\mathrm{Threshold}\;=\;\propto P_{noise},$$

where ∝ is the threshold factor and $P_{noise}$ is the estimated power of the noise. In this study, cell averaging of the CFAR [33] was implemented, therefore, the estimated power of the noise and the threshold factor can be achieved through the following calculations:

(2) $$P_{noise}=\frac{1}{N}\sum_{i=1}^{N}x_{i},$$

(3) ∝ = N(Pfa−1N−1),

where N is the number of training cells, $x_{i}$ is the sample in each training cell and Pfa is the desired false alarm rate. The desired false alarm rate should be chosen with precaution, because it affects the number of detected hits and the number of false alarms. With a higher $P_{fa}$, it is expected that more hits are registered, however, at the expense of a high rate of false hits. This is the opposite with a lower $P_{fa}$. Given this observation, it is of the utmost importance to choose a proper value of the $P_{fa}$. In this report, the $P_{fa}$ was set to 1% and each 1-s-long signal was segmented into 2000 cells for processing. The number of training and guard cells for each side of the CUT are both 10. The idea is that when the CUT’s power is higher than the threshold, the algorithm deduces that there is at least one target included in this region.

### 3.2. Hit Removal Process with OC-SVM

As mentioned previously, a hit is the way a sensor “sees” an AE event. Typically, at a sensor, only one direct hit can be achieved from its original event. However, in reality, more than one hit can be registered for the same event due to reflection, hit split, refraction, etc., especially when the specimen is small in size and damaged to a certain degree. In our test, the specimens undergo intense AE activity during the second phase of loading. With the progression of cracks, they no longer retain their original acoustic profile due to both external and internal damage. During this period, the number of hits is especially high. The number of non-direct hits through reflection and refraction is also expected to be significant in such a circumstance. Therefore, in order to get the most accurate number of hits to portray the deterioration, a process of hit removal is essential. Compared to the direct hit from a same source, the non-direct one(s) are delayed in time and less significant in amplitude. This, however, is yet to be distinguished with hits recorded from a more distant source. Therefore, to remove the unwanted hits without having to sacrifice the hits originating from distant events, we focus only on the ones that are very different from the rest (anomalies). Through this process, the DNN can learn to construct HI lines closer to the actual specimen state.

The support vector machine (SVM) was first introduced in 1992 by Vapnik V.N. et al. [33] and quickly became one of the most popular machine learning methods. Primarily, the SVM handles classification tasks using multidimensional-space hyperplanes for the separation of different classes. The reason behind its popularity and its great power with nonlinear separable data is the kernel trick. The kernel trick, in short, can make a non-linear problem into a linear one by projecting it to a higher dimension where it is linearly solvable.

As a natural extension of SVM, the purpose of one-class SVM (OC-SVM) fundamentally revolves around class separation, but the change is that the separation is harnessed to differentiate the “suspicious” observations (anomalies) from the rest of the data. This algorithm focuses on two aspects: 1—the estimation of a probability distribution function that is more likely to include the majority of data than the rest; 2—the decision rule which ensures the largest possible margin during the separation of these observations.

The original OC-SVM version by Schölkopf et al. [31] utilizes the kernel trick to capture regions in the input space where the probability density of the data points resides in a binary function and returns +1 in such a region, whereas −1 is returned for the rest. The quadratic programming minimization function of OC-SVM is not too much different from that of the original SVM:

(4) $$min_{w,b,\xi_{i}}\left(\frac{{\left|\left|w\right|\right|}^{2}}{2}+C\sum_{i=1}^{N}\xi_{i}-\rho \right),$$

which is subject to:

$$\begin{array}{ll} & w.\phi \left(x_{i}\right)\geq \;\rho -\xi_{i},\;\mathrm{for}\;\mathrm{all}\;i=1,\;2,\;3,\;\ldots ,\;N\\ & \xi_{i}\;\geq 0,\;\mathrm{for}\;\mathrm{all}\;i=1,\;2,\;3,\;\ldots ,\;N \end{array}$$

with $\xi_{i}$ being the slack variables that allow data points to be within the margin, *C* as the penalty parameter which characterizes the trade-off between the number of data points within the margin and how big the margin is.

The decision function is achieved after solving this minimization problem with Lagrange multipliers:

(5) $$f\left(x\right)=sign\left(\left(w.\phi \left(x_{i}\right)\right)-\rho \right)=sign\left(\sum_{i=1}^{N}\alpha_{i}K\left(x,x_{i}\right)-\rho \right),$$

Instead of the planar approach used by the original SVM, Tax and Duin‘s OC-SVM (SVDD) [32] focuses on a spherical approach. What can be obtained from this approach is a spherical boundary, which is characterized by a center A and a radius R. This approach’s minimization problem is as follows:

(6) $$min_{R,\;A}\left(R^{2}+C\sum_{i=1}^{N}\xi_{i}\right),$$

which is subject to:

$$\begin{array}{l} \left|\left|x_{i}-A\right|\right|\geq \;R^{2}+\xi_{i},\;\mathrm{for}\;\mathrm{all}\;i=1,\;2,\;3,\;\ldots ,\;N\\ \xi_{i}\;\geq 0,\;\mathrm{for}\;\mathrm{all}\;i=1,\;2,\;3,\;\ldots ,\;N \end{array}$$

Again, by using Lagrange multipliers, it can be simplified as follows:

(7) ||z−x||2=∑i=1Nαie−||z−xi||2σ2≥−R22+CR

**Figure 5:**
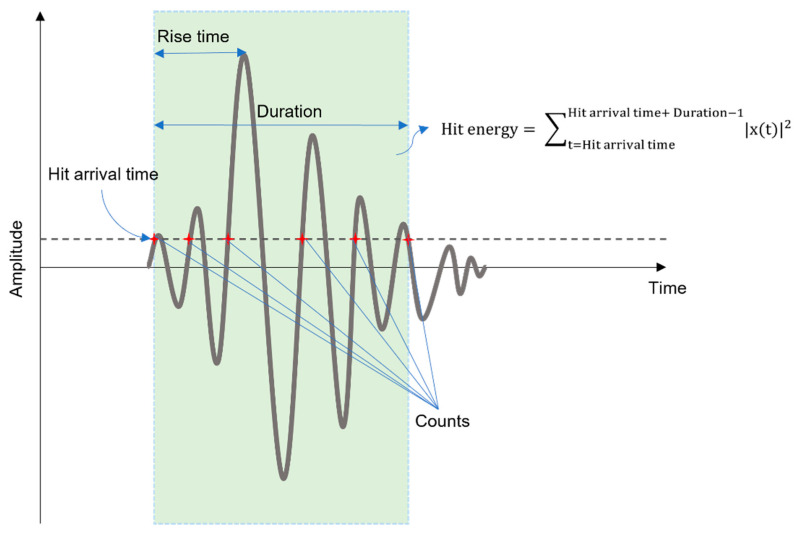
AE hit parameters.

In our study, the version of OC-SVM proposed by Tax and Duin is utilized along with the Radial Basis Function (RBF) kernel. The data of channels 5 to 8 from the three tests’ second phase are utilized for the training as one class. The trained OC-SVM is then utilized to seek for anomalies in the remaining set of data, which is later utilized as the training label for the DNN, which is to be discussed in the following section. Our OC-SVM considers hit arrival time, duration, hit energy, rise time of the hit, and hit counts for the process of anomaly detection. These characteristics of a hit are illustrated in Figure 5.

### 3.3. HI Constructor Using SAE-DNN

In order to get the HI lines that are later utilized for the RUL prediction, the process initiates with the construction of the DNN which outputs these curves. The DNN is designed so that it takes in the signal spectrum and outputs the HI in the range of [0, 1]. At the start, fast Fourier transform (FFT) is applied to each one-second segment of the signal, which returns 2.5 × 106 data points. However, since that number is undesirably large, the spectrum is divided into 2000 equal bands, each with an approximated energy that is calculated through the root mean square (RMS). This vector of size 2000 is fed to the stacked autoencoder (SAE) for the pre-training purpose. The encoder has three dense layers with diminishing sizes from 1000 to 200 to 10 with Xavier initialization and an exponential linear unit (ELU) activation function that are then utilized to process these data. Afterward, the decoder with three size-increasing dense layers from 200 to 1000 to 2000 takes in the encoder’s output for the reconstruction. The SAE’s regularization can be improved by adding dropout layers with a rate of 0.1 preceding the dense layers. Unlabeled signal spectra are harnessed as both inputs and outputs for SAE training along with Adam optimization and the fractions of masked zero at 0.1.

Following the training of the SAE, the encoder’s layers are reused as hidden layers in our DNN model, which also includes a logistic regression layer at the top. Fine-tuning is then performed in a supervised manner with an output size of one regarding the normalized number of AE hits in the range of [0, 1]. Since the number of hits represent how much damage the specimen takes at a moment, these values of normalized AE hit number can be considered the health indicator. The learning ability of high-level features from low-level ones of the SAE model is preserved by freezing the reused layers during DNN training, along with early stopping and checkpoint techniques to achieve the best parameters.

### 3.4. RUL Prognosis Using LSTM-RNN

Previously, the problem of vanishing and exploding gradients in RNN training had been a major problem. However, with the birth of LSTM, came a solution, especially for temporal sequences and long-range dependency models. What makes LSTM superior to the conventional RNN is the introduction of different gates and components, such as hidden units and memory cells, which allow the model to retain and process the information stored for a long time.

There are a total number of three main types of gates for LSTM: the input gate, forget gate, and output gate. The general idea of these gates is that they regulate the flow of information through storing, writing, and reading via gate opening or closing.

The forget gate $f_{\left(t\right)}$’s role is to determine whether to keep or dispose a piece of information. The current input $x_{\left(t\right)}$ and hidden state $h_{\left(t-1\right)}$ are fed to a logistic activation function, whose output represents the necessity of the information. The result $f_{\left(t\right)}$ equal to 0 indicates that the information is not needed and the gate is hence closed while it being 1 shows the opposite. Later, $f_{\left(t\right)}$ is utilized for an element-wise multiplication operation. Overall, the forget gate’s calculation is performed as follows:

(8) $$f_{\left(t\right)}=\sigma \left(W_{xf}x_{\left(t\right)}+W_{hf}h_{\left(t-1\right)}+b_{f}\right)$$

Subsequently, the evaluation of the new information is conducted before determining whether to store it in the internal state. The current input $x_{\left(t\right)}$ and previous hidden state $h_{\left(t-1\right)}$ are once again fed to a sigmoid activation function to achieve $i_{\left(t\right)}$ and then to a tanh function afterward to output $g_{\left(t\right)}$. This tanh layer, which can sometimes be referred to as an “input modulation gate”, constructs a candidate state vector $c_{\left(t\right)}$. The role of the input gate is to find the supplementation for the long-term state $c_{\left(t\right)}$ from $g_{\left(t\right)}$. $i_{\left(t\right)}$ and $g_{\left(t\right)}$ are achieved as follows:

(9) $$i_{\left(t\right)}=\sigma \left(W_{xi}x_{\left(t\right)}+W_{hf}h_{\left(t-1\right)}+b_{f}\right)$$

(10) $$g_{\left(t\right)}=tanh(W_{xg}x_{\left(t\right)}+W_{hg}h_{\left(t-1\right)}+b_{g})$$

Following the calculations in Equations (8)–(10), the current state is computed from the previous internal state $c_{\left(t-1\right)}$ as follows:

(11) $$c_{\left(t\right)}=f_{\left(t\right)}⊗c_{\left(t-1\right)}+i_{\left(t\right)}⊗g_{\left(t\right)}$$

Thereupon, the output gate $o_{\left(t\right)}$ decides which parts of the long-term state can be read and output through evaluation. The remaining state values are acquired by passing the internal state $c_{\left(t\right)}$ through a tanh layer and then by multiplication with the output of the sigmoid gate, which is computed by the equation below:

(12) $$y_{\left(t\right)}=h_{\left(t\right)}=o_{\left(t\right)}⊗tanh(c_{\left(t\right)}),$$

with:

(13) $$o_{\left(t\right)}=\sigma \left(W_{xo}x_{\left(t\right)}+W_{ho}h_{\left(t-1\right)}+b_{o}\right),$$

where W and b are the layer’s weight and bias, respectively.

Each data stream, which is a sequence of values along with time steps, can be considered a univariate time series. Each stream is utilized to train the RUL-predictor LSTM with segments of size 50. The segments are achieved by sliding a 50-value-long window with a step of 1 along the series. The model is adjusted such that it has to make a forecast at every time step rather than just the final one, which allows the loss to contain a term for every time step. This enables more error gradients to flow through the model, which leads to both faster training and stabilization at the same time [25]. When the data is assigned to a certain point in time, the predicted time step at the last moment is used as the 50th value in the next window for further computation.

In our model, the input layer is followed by two hidden LSTM layers of size 20. The dense output layer of size 1 harnesses a linear activation function. Both early stopping and checkpoints are similarly utilized during training, as with the previous DNN construction.

## 4. Result and Discussion

### 4.1. Hit Removal Result

**Figure 6:**
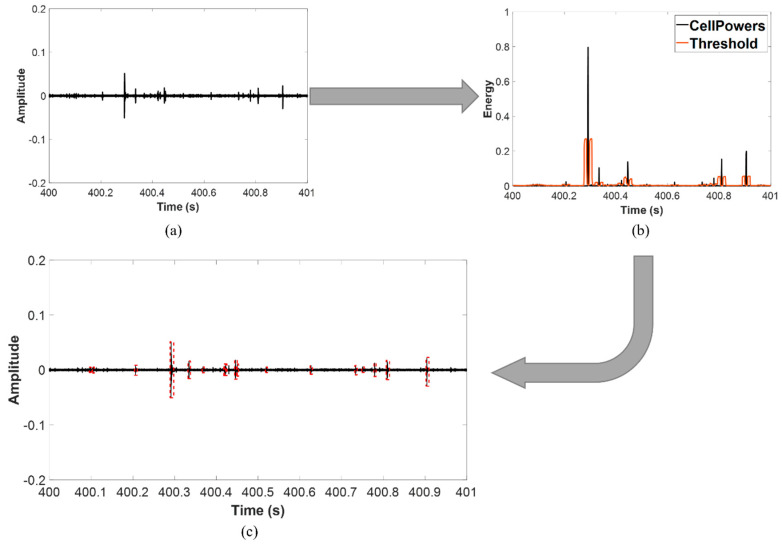
Application of the CFAR on sensor 8, specimen A during time steps 400–401. (**a**) Raw data, (**b**) Cell power versus threshold, (**c**) Detected hits.

Initially, the AE hits are detected by the CFAR algorithm. Figure 6 illustrates the result of hit detection from one sensor during the period between the 400th and 401st s.

After the OC-SVM model is trained with half of the data from channel 5 to 8, it is then harnessed to find anomalies in the remainder for later use in RUL prediction. The detailed result is given in Table 2.

By separating the anomalous hits from the rest, we can portray more accurately the specimen’s deterioration process. With the new set of data, the RUL predictor is expected to obtain the prognosis line closer to the actual HI curve, which is shown in the following section.

**Figure 7:**
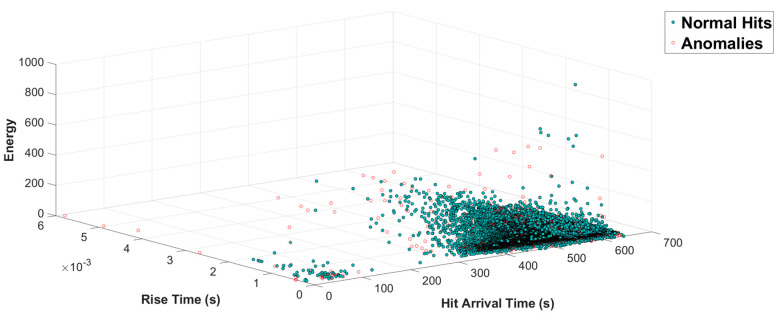
Visualization of normal versus anomalous hits in regard to the hit arrival time, rise time, and energy.

Through Figure 7, the result of such anomaly detection from channel 8 in regard to the hit arrival time, rise time, and energy can be witnessed.

### 4.2. HI Lines Construction and Prognosis

**Figure 8:**
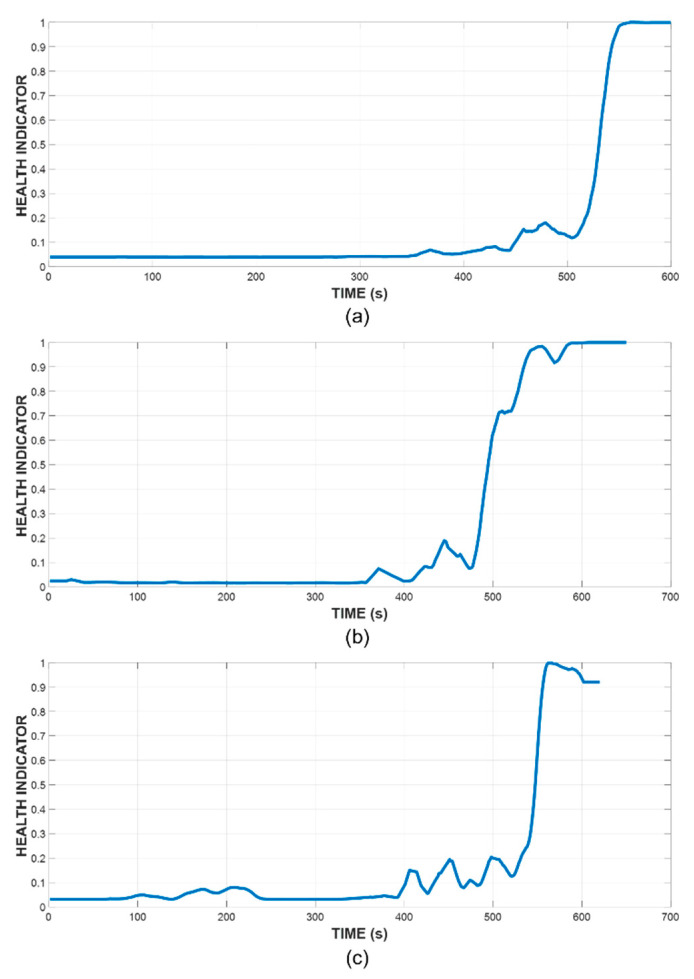
HI lines constructed by SAE-DNN (**a**) Specimen A, (**b**) Specimen B, (**c**) Specimen C.

The HI lines are obtained from the DNN after pretraining and fine-tuning processes. Figure 8 shows the example of result lines obtained from Sensor 1 over three tests.

RUL prediction priority is not identical at every moment during the loading process. At the earlier stage, the prediction is not yet of utmost importance due to the low probability of failure occurrence and lack of data. However, as time passes, the need for RUL prediction becomes increasingly important. Therefore, to highlight the prediction capability of our model, two major time steps at 350 and 450, which respectively mark the start of a minor and major crack, are utilized for RUL calculation. The error of prediction is calculated as follows:

(14) $$Error=absolute\left(T_{predicted}-T_{actual}\right),$$

with $T_{predicted}$ being the time of the predicted RUL and $T_{actual}$ being the time of the actual RUL.

**Figure 9:**
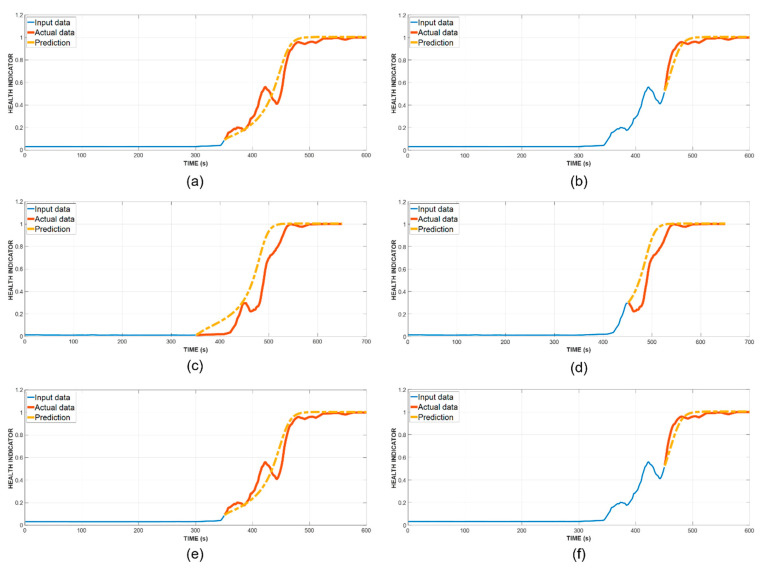
HI prediction results from sensor 8 at two different time steps during the three tests. (**a**) Specimen A at the 350th s. (**b**) Specimen A at the 450th s. (**c**) Specimen B at the 350th s. (**d**) Specimen B at the 450th s. (**e**) Specimen C at the 350th s. (**f**) Specimen C at the 450th s.

The specimen’s health index lies within [0, 1] range, with a higher value indicating a higher level of damage sustained by the specimen. In our study, we mark the end of useful lifetime when this value surpasses 0.95, hence, the RUL is calculated accordingly. The prediction result from sensor 8 in the three tests is shown in Figure 9.

In the first test, our prediction obtained the average prediction error of 25 at the 350th s and 19 at the 450th s across four sensors, while, in the absence of the hit removal process, they are higher at 32 and 21, respectively. For the second test, we similarly achieved a better prediction error of 37 and 28, in comparison with 41 and 34 without hit removal before the training of DNN. The final test showed a similar result for the first prediction, however for the second one, our prediction still offered a better error at 21 rather than 24 when the DNN was trained with the unfiltered hit number. For further details concerning the comparison between our method, LSTM-RNN with anomalous hit removal (AHR), versus others, Table 3 is presented below.

## 5. Conclusions

In this paper, we presented a scheme of remaining useful life (RUL) prognosis from raw acoustic emission (AE) signals. From the raw signals, a health indicator (HI) constructor based on deep neural network pretrained and fine-tuned with stacked autoencoder (SAE-DNN), whose training is performed with AE hits as the label. We also proposed that the AE hits utilized for DNN training are processed through a hit removal process in advance, which filters out the ones that stray the furthest from the other hits. With the aforementioned process, the DNN can construct better HI lines which later are a vital part of RUL prediction.

After the DNN constructs HI lines from raw AE data, these lines are fed into the long short-term memory recurrent neural network (LSTM-RNN) for prognosis. To verify the validity of our method, we performed two RUL estimations for each of the three tests at the passing of the 350th and 450th s, with regard to the fact that they mark the start of micro and macro fractures, respectively. The results obtained from the proposed method show significant improvement over the reference methods, such as gated recurrent unit recurrent neural network (GRU-RNN) and recurrent neural network (RNN). Furthermore, a performance comparison is made between the proposed scheme and a similar scheme without the hit removal process, which also indicates notable amelioration in RUL prediction.

For upcoming studies, this method can be further extended to other concrete structures such as walls, buildings, etc. Other approaches to RUL prognosis instead of an LSTM-RNN are also considered for future work.

## Figures and Tables

**Table 1:** Experimental specifications.

No.	Parameters	Details
1	Number of sensors	8
2	Type of sensor	R3I-AST
3	Sampling frequency	5 MHz
4	Concrete compressive strength	24 MPa
5	Beam size	150 × 300 × 2400 mm
6	Steel rebar	D16 (SD400)
7	Load velocity	1 mm/s
8	Acquisition time	Varying

**Table 2:** Hit removal result.

Channel	Total Number of Hits	Number of Anomalies (Per Total Number of Hits)
5	16,684	1497 (8.97%)
6	24,067	2658 (11.08%)
7	21,475	2065 (9.62%)
8	27,052	2727 (10.08%)

**Table 3:** The proposed method’s result in comparison with LSTM-RNN without AHR, GRU-RNN, and Simple-RNN.

Channel	Method	Total Number of Hits	Number of Anomalies (Per Total Number of Hits)
A	LSTM-RNN with AHR	25 ± 7	19 ± 6
	LSTM-RNN without AHR	32 ± 3	21 ± 4
	GRU-RNN	36 ± 4	32 ± 5
	Simple-RNN	81 ± 6	61 ± 8
B	LSTM-RNN with AHR	37 ± 5	28 ± 4
	LSTM-RNN without AHR	41 ± 7	34 ± 5
	GRU-RNN	43 ± 6	37 ± 7
	Simple-RNN	95 ± 11	89 ± 8
C	LSTM-RNN with AHR	36 ± 3	21 ± 3
	LSTM-RNN without AHR	36 ± 3	24 ± 3
	GRU-RNN	39 ± 7	32 ± 3
	Simple-RNN	88 ± 4	68 ± 7

## Data Availability

The data is available upon the request.
